# Improving the Understanding and Managing of the Quality of Life of Patients With Lung Cancer With Electronic Patient-Reported Outcome Measures: Scoping Review

**DOI:** 10.2196/46259

**Published:** 2023-05-03

**Authors:** Abel García Abejas, Adrià Serra Trullás, Maria Ana Sobral, Daniel Canelas, Fábio Leite Costa, Àngels Salvador Verges

**Affiliations:** 1 Intra-hospital Palliative Care Team Hospital Lusíadas Lisbon Portugal; 2 Bioethics and Medical Ethics Faculdade de Ciências da Saúde Universidade da Beira Interior Covilhã Portugal; 3 NEBUBI (PalUBI-Research Group) Faculdade de Ciências da Saúde Universidade da Beira Interior Covilhã Portugal; 4 Department of Orthopedic Surgery and Traumatology Hospital Clínic Barcelona Spain; 5 Intra-hospital Palliative Care Team Hospital Fernando da Fonseca Amadora Portugal; 6 Department of Telemedicine Hospital CUF Lisbon Portugal; 7 General Practice and Family Medicine Unidade de Saúde Familiar Afonsoeiro Montijo Portugal; 8 Palliative Care Team Unidade de Cuidados Continuados Integrados Francisco Marques Estaca Júnior Alhos Vedros Portugal; 9 General Practice and Family Medicine Unidade de Saúde Familiar Moscavide Loures Portugal; 10 Innohealth Academy Barcelona Spain; 11 Hiberiae Societas Telemedicinae et Telesanitas Barcelona Spain

**Keywords:** lung cancer, electronic patient-reported outcome measures, ePROMs, health-related quality of life, sickness impact profile, quality improvement, review

## Abstract

**Background:**

Electronic patient-reported outcome measures (ePROMs) are essential to clinical practice and research. The growth of eHealth technologies has provided unprecedented opportunities to collect information systematically through ePROMs. Although they are widely used in scientific research, more evidence is needed to determine their use and implementation in daily clinical practice. For example, when diagnosed, patients with lung cancer are at an advanced stage of the disease. This entails tremendous burden because of high mortality and losses in the different dimensions of the human being. In this case, monitoring symptoms and other outcomes help improve the patient’s quality of life.

**Objective:**

ePROMs offered unprecedented opportunities to collect information systematically. Our goal was to demonstrate that ePROMs are more useful in controlling patient symptoms, lung cancer, and overall survival than their alternatives, such as nonelectronic PROMs.

**Methods:**

This exploratory review considered articles published between 2017 and 2022 identified through searches of PubMed, Scopus, Cochrane, CINAHL, and PsycINFO. We found 5097 articles; after removing the duplicates, we reduced them to 3315. After reading the summary, we were left with 56. Finally, after applying the exclusion criteria, we reviewed 12. The 5-step framework by Arksey and O’Malley was used to refine the initial search results with the following research questions: Do ePROMs help physician-patient communication? To what extent do they improve decision-making? Are institutions and their digitization policies barriers to or facilitators of this process? and What else is needed for routine implementation?

**Results:**

This review included 12 articles. We found that ePROMs are an integrative and facilitative communication tool, highlighting their importance in the relationship between palliative care and medical oncology. ePROMs help assess patient symptoms and functionality more accurately and facilitate clinical decision-making. In addition, it allows for more precise predictions of overall patient survival and the adverse effects of their treatments. The main institutional obstacles are the initial investment, which can be costly, and the data protection policy. However, enablers included better funding through the development of telemedicine, support from institutional leaders to overcome resistance to change, and transparent policies to ensure the safe and secure use of ePROMs.

**Conclusions:**

Routine collection of remote ePROMs is an effective and valuable strategy for providing real-time clinical feedback. In addition, it provides satisfaction to patients and professionals. Optimizing ePROMs in patients with lung cancer leads to a more accurate view of health outcomes and ensures quality patient follow-up. It also allows us to stratify patients based on their morbidity, creating specific follow-ups for their needs. However, data privacy and security are concerns when using ePROMs to ensure compliance with local entities. At least four barriers were identified: cost, complex programming within health systems, safety, and social and health literacy.

## Introduction

### Background

Lung cancer is the second most common cancer affecting men and women. Approximately 13% of all new cancers are lung cancer. Approximately 236,740 new cases were diagnosed in the United States alone in 2022.

Lung cancer accounts for approximately 25% of all cancer-related deaths in the United States. However, death rates from this disease have declined by 54% since 1990 in men and by 32% since 2002 in women. From 2015 to 2019, death rates in men with lung cancer decreased by 5% per year and death rates in women with lung cancer decreased by 4% per year. Research suggests that these declines are due to fewer people smoking, more people quitting smoking, and advances in diagnosis and treatment [1].

Incidence and mortality are highest in high-income countries such as those in Europe, North America, and Australia [2]. Survival is poor, with a median 5-year survival rate of 15% [3].

Lung cancer mainly occurs in the older adult population. Most people diagnosed with it are aged ≥65 years or older and few are younger than 45 years. The average age of patients at the time of diagnosis is approximately 70 years.

Initially, lung cancer is symptom free but nonspecific symptoms such as cough, pain, dyspnea, and hemoptysis appear later. Because of the initial symptom-free course, lung cancer is often diagnosed at an advanced stage, and its symptoms burden affects the quality of life [4]. In addition, survival-enhancing chemotherapy often has indicative adverse effects that affect quality of life [5].

Health-related quality of life (HRQoL) includes several domains that consider a patient’s overall perception of the impact of the disease or treatment on the physical, psychological, and social aspects of life [6].

Patient-reported outcome measures (PROMs) are outcomes related to their health status and are reported directly by the patient [7]. PROMs are tools used to assess patients’ views of their health status, including HRQoL, symptom status, physical function, and mental health [8]. They can be used in patient-physician communication and clinical decision-making. In addition, the increasing use of PROMs has contributed to the paradigm shift from disease-centered care to patient-centered care [9]. Randomized controlled trials comparing PROM-directed follow-up with usual care have demonstrated that integrating PROMs into care pathways is associated with improved symptom control, survival, and reduced emergency department attendance and hospitalizations [10-12]. Although electronic PROMs (ePROMs) monitoring is easily an essential part of value-based health care, to enable benchmarking with patients and colleagues from other hospitals, a set of standardized patient-centered outcome indicators should be defined in nonsmall cell lung cancer, which also makes digital monitoring of the quality of life of patients more manageable [12].

ePROMs can help clinicians to better understand patients’ overall care and monitor their progress. The data collected can also be used in research to help identify risk factors and better understand the outcomes associated with lung cancer. ePROMs can also be used to develop algorithms that can create individualized treatment plans for each patient and enable better tracking of outcome changes [7,10,11].

The inclusion of PROMs as end points in clinical trials is encouraged by the Food and Drug Administration, the European Medicines Agency [13], and scientific societies such as the European Society for Medical Oncology [14].

### Use in Routine Clinical Practice

PROMs provide essential information about the impact of a disease or treatment on the patient while complementing other, more traditional outcome information, such as survival and time to symptom resolution [15].

With the transition to patient-centered care, there is a growing interest in the routine application of PROMs in clinical settings. However, implementing PROMs is challenging for patients, clinicians, and institutions wishing to use them [16]. Although PROMs have grown in popularity and are increasingly being used, the pioneers in PROM collection are mainly the United Kingdom, Sweden, Australia, parts of the United States, and Canada.

### Objectives

Our objective was to understand the challenges of implementing ePROMs to improve HRQoL through articles published in the last 5 years, posing four research questions:

1. Do ePROMs help physician-patient communication?
2. To what extent do they improve decision-making?
3. Are institutions and their digitalization policies barriers or enablers?
4. What else is needed for their routine application?

## Methods

### Study Design

We chose the scoping review methodology because it is more exploratory and less methodological than systematic reviews, which are essential to meet the study’s objectives. The research strategy was developed following the Arksey and O’Malley methodological framework [17], which proposes a transparent 5-stage process for replicating research strategies to increase the reliability of the results. The first stage clarifies and links the purpose of the study and the research questions; the second stage balances feasibility with the comprehensiveness of the research process; the third stage includes study selection; the fourth stage involves data mapping; and the fifth stage summarizes the findings.

### Clarifying and Linking the Purpose to the Research Questions

This study aimed to review the literature to assess whether ePROMs help us understand the needs of patients and therefore make clinical decisions in this regard.

**Figure 1:**
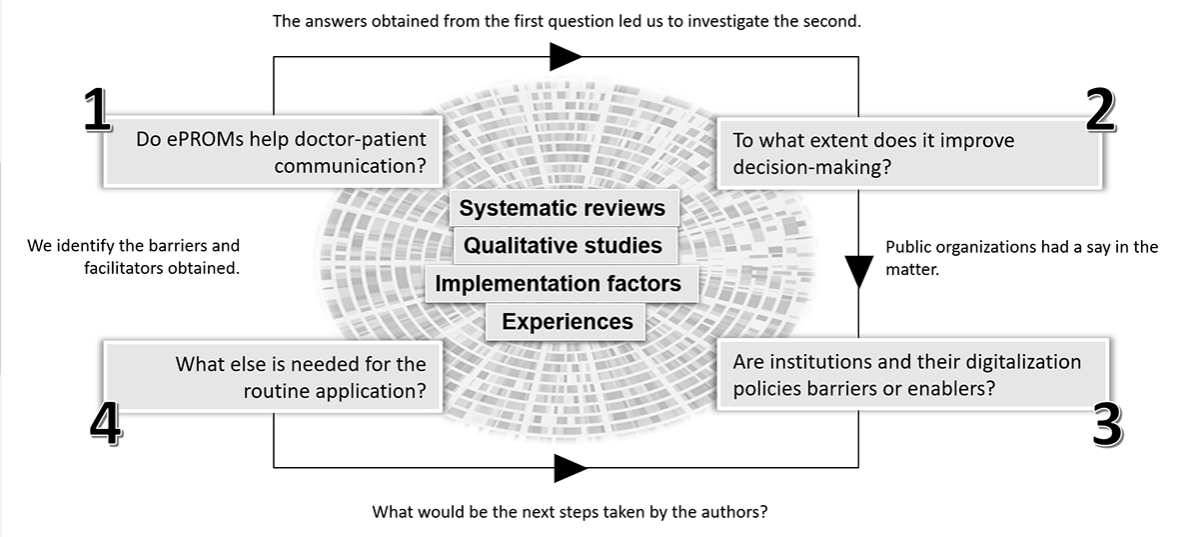
Conceptual framework of the scoping review. In the center of the image is the grouping of the selected items. ePROM: electronic patient-reported outcome measure.

After determining the research questions, we developed a conceptual framework to define and map the critical concepts and identify research gaps that could hinder their use (Figure 1). The conceptual framework guided the analysis and systematic presentation of the summarized data. The 4 research questions constituted the main branches of the framework, and the extracted data were classified into the 4 types of articles chosen (systematic reviews, qualitative studies, implementation factors, and experiences) to relate the opinions of the authors to our research questions.

### Balancing Feasibility With Process Breadth

A literature search was conducted between January and December 2022 and included the Scopus, Web of Science, PubMed, CINAHL, and PsycINFO databases. Correct key terms are critical for facilitating maximum coverage of the related research literature [18]. Medical Subject Headings terminology (“PROMs” AND “outcomes” AND “cancer” AND “lung”) was used to increase the sensitivity of the search. We also examined the reference list of each article. In addition, we expanded our search by adding the following terms: patient-reported outcomes and quality improvement.

### Selection of Articles

**Figure 2:**
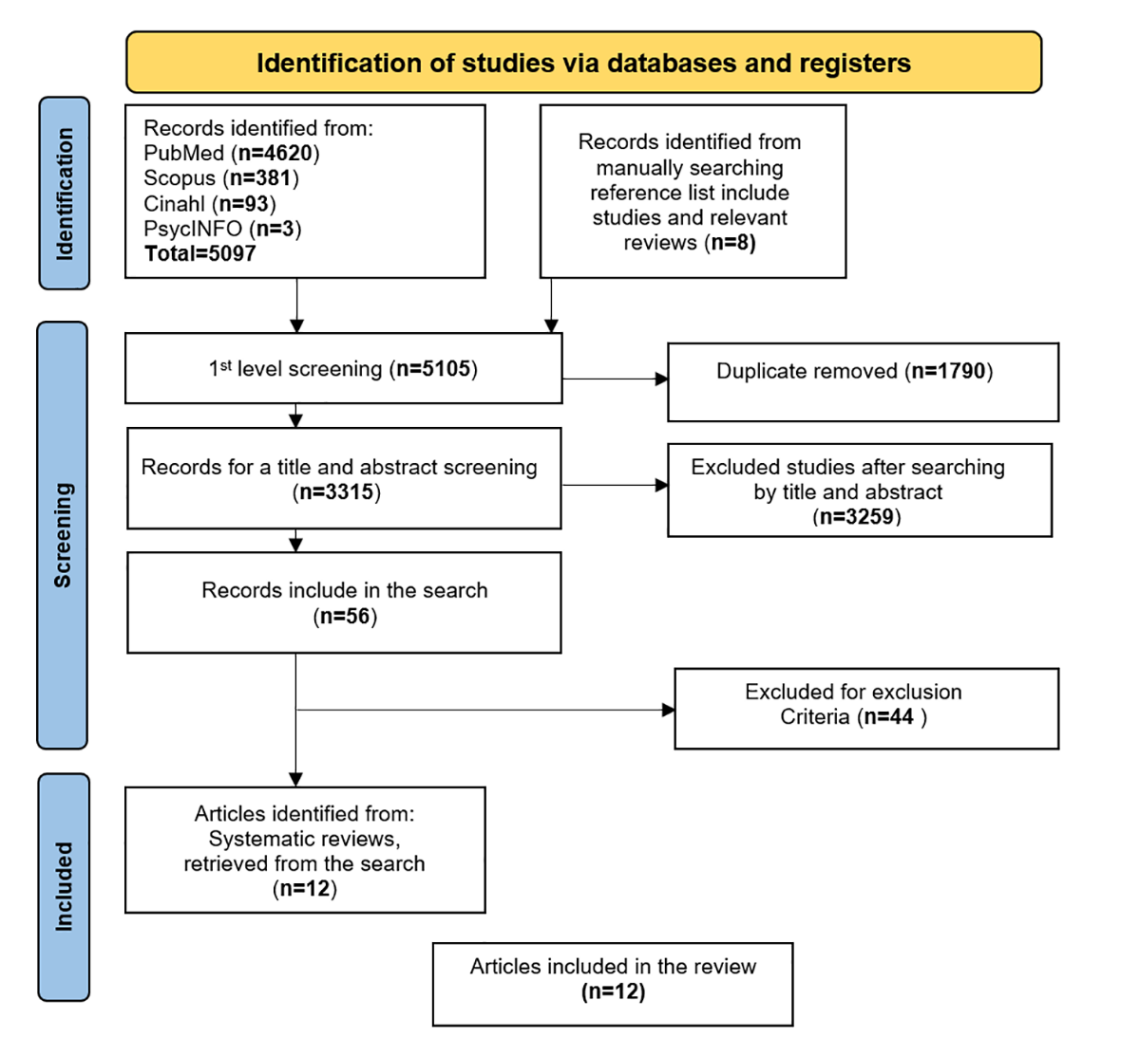
Reporting PRISMA (Preferred Reporting Items for Systematic Reviews and Meta-Analyses) flow diagram for the scoping review process.

Scoping reviews [19] map the underlying concepts; therefore, defining *methods* is essential, as with other types of knowledge synthesis [20]. In 2015, the Joanna Briggs Institute published methodological guidelines [21]. This methodology involves incorporating a checklist to increase the transparency of the method, judge validity and reliability, and appropriately manage the search [22]. Among the existing forms of presentation, we focused on the revised and expanded PRISMA-RR (Preferred Reporting Items for Systematic Reviews and Meta-Analyses for rapid reviews) [23], which illustrates the transparency of the item selection (Figure 2).

## Results

### Inclusion and Exclusion Criteria

All the articles that met the inclusion criteria were subjected to information extraction. In addition, the inclusion criteria and the validity of the identified instruments were assessed (Table 1).

**Table 1:** Inclusion and exclusion criteria.

Criteria	Inclusion	Exclusion
Keyword	Lung cancer and rare diseases	Other types of diseases
Language	English	Not written in English
Year of publication	2017-2022	Articles that were published before 2017
Type of published journal	Peer reviewed	Articles that were not peer reviewed
Ethics permission	Ethics permission obtained	Articles without approved ethics permissions

### Extraction and Graphical Representation of Results

The collected articles were organized by author, title, year, country, and type of article. The selected articles included 42% (5/12) from the United Kingdom, 17% (2/12) from the Netherlands, and 8% (1/12) from each of the following country: Germany, Canada, Australia, the United States, and Italy. All studies reviewed contributed to understanding the complexity of applying ePROMs in routine clinical practice (Table 2).

**Table 2:** Selected articles.

Study	Title	Year	Country	Type of article
Crockett et al [24]	The Routine Clinical Implementation of Electronic Patient-reported Outcome Measures (ePROMs) at The Christie NHS Foundation Trust	2021	United Kingdom	Presentation of experiences
Aiyegbusi et al [25]	Patient and clinician opinions of patient reported outcome measures (PROMs) in the management of patients with rare diseases: a qualitative study	2021	United Kingdom	Qualitative studies
Scheibe et al [26]	Implementation of patient-reported outcome assessment in routine cancer care: A systematic review of multicentric programs in Europe	2020	Germany	Systematic reviews
Al Sayah et al [27]	A multi-level approach for the use of routinely collected patient-reported outcome measures (PROMs) data in healthcare systems	2021	Canada	Factors for implementation
Carlton et al [28]	An emerging framework for fully incorporating PI^a^ into PROMs	2020	United Kingdom	Factors for implementation
Nordan et al [29]	Implementing electronic patient reported outcomes measurements: challenges and success factors	2018	United States	Factors for implementation
Girgis et al [30]	Stepping into the real world: a mixed-methods evaluation of the implementation of electronic patient reported outcomes in routine lung cancer care	2022	Australia	Qualitative studies
Convill et al [31]	The Role of Electronic Patient-Reported Outcome Measures in Assessing Smoking Status and Cessation for Patients with Lung Cancer	2022	United Kingdom	Presentation of experiences
Liao et al [32]	Prognostic value of patient-reported outcome measures (PROMs) in adults with non-small cell Lung Cancer: a scoping review	2022	United Kingdom	Systematic reviews
Bouazza et al [33]	Patient-reported outcome measures (PROMs) in the management of lung cancer: A systematic review	2017	The Netherlands	Systematic reviews
Brunelli et al [34]	Knowledge, use and attitudes of health care professionals towards patient-reported outcome measures (PROMs) at a comprehensive cancer center	2022	Italy	Qualitative studies
Meirte et al [35]	Benefits and Disadvantages of Electronic Patient-reported Outcome Measures: Systematic Review	2020	The Netherlands	Systematic reviews

^a^PI: public involvement.

### Communication of Results

The articles were classified according to study design:

- Systematic reviews
- Qualitative studies
- Factors for implementation
- Presentation of experiences

Table 3 summarizes the authors’ contributions to the first research question. This grouping allowed us to identify an approach based on the line of inquiry.

ePROMs are an integrating and facilitating tool for communication, highlighting their importance in the relationship between palliative care and medical oncology. In addition, they facilitate the knowledge and management of outcomes obtained from patients so that patients, relatives, physicians, and even administrations or institutions have access to these data and can compare them, thus facilitating communication, improving quality of life, and generating lower costs.

We must emphasize that this tool, used in patients with rare diseases subject to palliative care, can facilitate communication and the physician-patient relationship. This specific way of obtaining information enhances, as we have seen, communication and therefore drives more timely care depending on the level of need. In oncology, there is no established routine for this collection of ePROMs; therefore, its application in this area is subject to study. Furthermore, its implementation improves clinical communication with better-structured data that facilitates interpretation. The patients who participated in some of the implementation programs confirmed that they were helpful, were easy to learn and use, and facilitated their communication with the health care team. We are also aware that their use automatically improves this process.

**Table 3:** Do electronic patient-reported outcome measures (ePROMs) help to improve physician-patient communication?

Study		Contribution
**Systematic review**		
	Scheibe et al [26], 2020	Outcome measurement programs allow for two primary purposes: (1) tracking an individual patient’s outcome to aid treatment decision-making and (2) use in quality improvement initiatives, including provider benchmarking.
	Liao et al [32],2021	Their study measures ePROMs as prognostic factors for survival outcomes in patients with lung cancer.
	Bouazza et al [33], 2017	ePROMs improve the process of care, resulting in better patient outcomes. For data comparison to being meaningful and reliable, it must be corrected sufficiently to account for differences in caseness. Caregivers can also evaluate data and compare information from others. It would be cost-effective, as there would be fewer medical errors and unnecessary costs owing to the high quality of care.
	Meirte et al [35], 2020	Overall, ePROMs are preferable to paper-based methods, improve data quality, result in similar or faster completion time, decrease costs, and facilitate clinical decision-making and symptom management. In addition, most patients exposed to ePROMs found it easy to learn and use, would recommend it to other patients, and would like to continue using it.
**Qualitative studies**		
	Aiyegbusi et al [25], 2021	ePROMs in managing patients with rare diseases can facilitate patient-centered care by promoting patient-physician communication, highlighting aspects of HRQoL^a^ that are important, and encouraging participation in their care.
	Girgis et al [30], 2022	Patient-reported outcome measures, ePROMs, via the internet make it easier for patients to report issues of concern to their care team, which can drive timely care based on the level of need.
	Brunelli et al [34], 2022	Routine use of ePROMs is considered an essential indicator of integration between oncology and palliative care. In addition, patients perceive ePROMs as relevant, easy to use, and helpful in describing their health conditions.
**Factors for implementation**		
	Crockett et al [24], 2021	ePROMs are associated with significant benefits to patient care and improved communication, increased patient and physician satisfaction, and increased consultation efficiency because of the availability of patient responses before consultations.
	Convill et al [31], 2022	An independent questionnaire, such as the ePROM, would be well suited to help identify patients who still smoke.
**Presentation of experiences**		
	Al Sayah et al [27], 2021	ePROMs improve clinical communication by allowing data to be presented in a structured way, facilitating their interpretation.
	Carlton et al [28], 2020	ePROMs measures are expected to improve patient-physician communication. However, although ePROMs measures have much potential, research shows that applying PROMs^b^ in clinical practice only automatically promotes patient engagement or improves communication.
	Nordan et al [29], 2018	Determining how to collect and use PROMs remains an area of debate and, in some cases, frustration. However, obtaining them would provide a clearer understanding of needed care pathways, treatment outcomes, and their improvement following medical or surgical intervention, which are often absent from our current health care processes. While PROMs are not new, the ability to collect, communicate, and use data electronically has become more relevant in recent years.

^a^HRQoL: health-related quality of life.

^b^PROM: patient reported outcome measure.

As this method becomes more familiar, physician-patient communication can be improved in the future.

Table 4 summarizes the authors’ contributions to the second research question. The collection of ePROMs helps better assess patient symptoms and functionality with greater accuracy and facilitates clinical decision-making. Measuring prognostic factors allows for a broader understanding and prediction of treatments’ toxic effects and survival, enabling physicians and patients to stop adverse treatments and make decisions earlier. According to various published studies, using ePROMs facilitates this decision-making because their recording can generate alerts, allowing health care workers to manage patients’ needs on time. With the collection of ePROMs, there is generally an improvement in patient-centered care and the entire decision-making process throughout the disease and treatment. In the pandemic and with the evolution of “digital health care,” this field has become a suitable and flexible alternative with the potential to soon become the standard of care. With these positive indicators, the current momentum demonstrates that models of implementation of this data collection system can add value to health care. The studies conclude that it improves the decision-making process and enhances dialogue and the depth of conversations, which is a factor of approximation in the physician-patient relationship. It improves feedback and facilitates better interpretation of the disease process, including improving survival and associated costs.

**Table 4:** To what extent do they improve decision-making?

Study			Contribution
**Systematic review**			
	Scheibe et al [26], 2020	PROMs^a^ help health care professionals assess patients' symptoms and function more accurately and assist them in clinical decision-making. Recent studies have also shown improved survival when patients with cancer are monitored with PROMs.	
	Liao et al [32], 2021	Like the measurement of prognostic factors, decision-making is a consequence of the observed factors. This warrants a more significant effort to predict a broader range of outcomes besides survival, such as treatment response, toxicity, and early treatment discontinuation, which would better assist patients and physicians in making complex treatment decisions.	
	Bouazza et al [33], 2017	There is increasing interest in the routine use of PROMs in daily practice, which positively affects patient communication, mutual decision-making, and patient monitoring and management.	
	Meirte et al [35], 2020	The role of ePROMs^b^ in symptom management and decision-making was recognized in multiple studies, which reported that electronic self-reporting of symptoms was necessary for clinical decision-making. In addition, automated data collection and processing through ePROMs can generate automatic alerts for health care professionals.	
**Qualitative studies**			
	Aiyegbusi et al [25], 2021	Systematic collection of ePROMs can help clinicians monitor patients’ symptoms, identify unmet needs and concerns, prioritize and tailor treatment to each patient's needs, and ultimately improve the quality of patient care.	
	Girgis et al [30], 2022	This study demonstrated that the use of ePROMs improved outcomes in the following domains: (1) the majority of eligible patients completed scheduled assessments; (2) patient concerns were identified at each assessment, and care coordinators reviewed and acted on nearly all of them, including making significantly more referrals to allied health services; (3) patients who completed assessments regularly were less likely to present to the cancer assessment unit with problematic symptoms, suggesting that ePROMs identified patient concerns early and this led to a timely response to concerns; and (4) staff training and engagement were high, and staff reported increased confidence in asking patients to complete assessments. In conclusion, the successful implementation of the ePROMs system in routine care could pave the way for redefining models of care that leverage the capabilities of automated web-based strategies and the involvement of staff from multiple disciplines in the implementation processes.	
	Brunelli et al [34], 2022	On the basis of the generally positive attitude of health care professionals, routine implementation of ePROMs can be promoted, as they aid in decision-making, as long as adequate resources and training are provided.	
**Factors for implementation**			
	Crockett et al [24], 2021	The use of ePROMs can help personalize patient care pathways, including the frequency or type of clinical review (eg, a face-to-face visit, phone call, or video call). This strategy has the potential to save unnecessary hospital visits for some patients, free up clinical capacity, and realize health economic benefits. To the best of our knowledge, The Christie NHS^c^ Foundation Trust is the first center in the United Kingdom to introduce ePROMs into the standard setting on a large scale for patients with cancer.	
	Convill et al [31], 2022	ePROMs may represent an efficient and accurate means of collecting and analyzing patient smoking information.	
**Presentation of experiences**			
	Al Sayah et al [27], 2021	Electronic databases can aid decision-making: it minimizes clinical and administrative burden, ensures timely feedback of PROM scores to clinicians and patients, and allows for proper presentation of data to facilitate interpretation.	
	Carlton et al [28], 2020	Some PROMs can be used to generate quality-adjusted life-years for use in economic evaluations of treatments.	
	Nordan et al [29], 2018	Incorporating ePROM ratings into the clinical process helps the patient and physician engage in a more relevant, patient-centered discussion. It increases the depth of conversations, enabling shared decisions about treatment possibilities. This capability and the ability to track the assessments of an individual patient, physicians, or a department led to greater acceptance by patients, physicians, and administrators.	

^a^PROMs: patient-reported outcome measures.

^b^ePROMs: electronic patient-reported outcome measures.

^c^NHS: National Health Service.

In Table 5, referring to the third research question, the author’s comments are as follows: the ePROMs are already being implemented in different areas, such as food and pharmaceuticals, as revealed by studies with benefits that are not immediate but long term [36]. However, the leading institutional barriers are the initial investment, which can be costly, and the data protection policy. In addition, the lack of time for proper care and literacy is considered a bidirectional barrier. On the other hand, factors such as patient reminders, a sense of self-control, and physician enthusiasm may be facilitating factors.

**Table 5:** Are institutions and their digitalization policies barriers or enablers?

Study			Contribution
**Systematic reviews**			
	Scheibe et al [26], 2020	For institutions to decide when planning PROMs^a^ programs, they should include the choice of instruments to be measured, mode of administration, and provision of feedback from all involved, among other elements (eg, where or on the web or paper).	
	Liao et al [32], 2021	It does not address this issue.	
	Bouazza et al [33], 2017	PROMs can be used as a performance indicator for health care institutions and organizations. The inclusion of PROMs as end points in clinical trials is encouraged by the FDA^b^, the EMA^c^, and scientific societies such as the EMSO^d^.	
	Meirte et al [35], 2020	Some of the barriers observed in the development of policies of the institutions are the need for more attention given to data protection, the technical difficulties of implementation, and the initial economic investment.	
**Qualitative studies**			
	Aiyegbusi et al [25], 2021	Institutions bring potential facilitators, such as patient reminders, clinician enthusiasm, and computer-based fitting tests, but also potential barriers, such as the lack of awareness, time constraints, and patient literacy and access. There are also practical considerations for implementation such as administrative issues, access to patient data, response to ePROMs^e^ data, and patient issues. For example, time constraints during consultations could prevent clinicians from acting on ePROMs results, which could hinder their use. Patients’ level of computer literacy; language; and access to the internet, computer, or telephone were other potential barriers identified.	
	Girgis et al [30], 2022	Health services are increasingly incorporating ePROMs to inform person-centered care and evaluate services. Telehealth, web-based care, and long-term follow-up are potentially viable alternative and complementary care models for the growing demand.	
	Brunelli et al [34], 2022	The authors suggest that more than cultural and scientific developments may be required for successful implementation and that organizational and resource allocation intervention may be equally important.	
**Factors for implementation**			
	Crockett et al [24], 2021	In January 2019, The Christie, a large tertiary cancer hospital in the United Kingdom, launched an ePROM service (“MyChristieMyHealth”) that integrated ePROM questionnaires into care pathways for adult patients with lung, head, and neck cancer and patients treated with proton beam therapy. We have learned that a dedicated team is necessary to ensure implementation and maximize the completion of ePROMs. Christie employs 2 patient outcomes coordinators who contact patients before receiving their first ePROM invitation to inform them of the service. They are also responsible for contacting patients who still need to complete follow-up ePROMs and supporting them. For this service to become a reality, a review of ePROM responses by clinical teams should be included in work plans.	
	Convill et al [31], 2022	Data from PROMs are used primarily in 2 ways: informing individual patient care and informing health services or facilitating policy development.	
**Presentation of experiences**			
	Al Sayah et al [27], 2021	International initiatives, such as the ICHOM^f^ and the OECD^g^ PaRIS^h^ have accelerated the movement toward routine measurement of PROMs in health systems.	
	Carlton et al [28], 2020	Christie employs 2 patient outcomes coordinators who contact patients before receiving their first ePROM invitation to inform them of the service. They are also responsible for contacting patients who still need to complete follow-up ePROMs and supporting them. For this service to become a reality, a review of ePROM responses by clinical teams should be included in work plans.	
	Nordan et al [29], 2018	Naturally, implementing ePROMs proved more difficult in some specialties than in others. In addition, the history of some employees with previous attempts to implement and collect ePROMs created barriers to implementation. Patient acceptance of remote capture of questionnaires was also a challenge. Finally, there were concerns about data security. Therefore, time and energy must be allocated to ensure compliance with the committee framework and organizational policies and procedures.	

^a^PROMs: patient-reported outcome measures.

^b^FDA: Food and Drug Administration.

^c^EMA: European Medicines Agency.

^d^EMSO: European Society for Medical Oncology.

^e^ePROMs: electronic patient-reported outcome measures.

^f^ICHOM: International Consortium for Health Outcomes Measurement.

^g^OECD: Organisation for Economic Co-operation and Development.

^h^PaRIS: Patient-Reported Indicator Surveys.

The large-scale experience implemented in the National Health Service indicates that a specialized and sensitized group is necessary for implementing these measures, such as 2 specialists in outcome management. On the other hand, the information collected allows for individual patient benefits as well as the collection of sufficient data to create institutional policies.

Initiatives such as those of the International Consortium for Health Outcomes Measurement and the Organisation for Economic Co-operation and Development have promoted the implementation of these policies, which is a positive aspect. However, organizational and institutional policies are required to implement the ePROM system successfully.

Cultural, institutional, and individual barriers require specific training to change the paradigm. However, the results are promising, as the exposed patients are usually satisfied with handling digital tools, whether through the web or applications.

Table 6 shows the authors’ conclusions on its routine application: (1) It is necessary to define what is to be measured, what type of patients will be exposed, and in which services these policies can be implemented. (2) Changing work culture is a barrier to implementation and creates resistance to change. The difficulty that some patients may face in accessing technology (without minimizing the capabilities of older adults) is an important point, as is the lack of cybersecurity confidence. (3) The use of ePROMs has been established in scientific research, particularly in drug studies, although it is still far from its broader use in clinical reality and routine use. Further investigation of their implementation is necessary for greater confidence and ability to change clinicians’ work. (4) Physicians and patients must maintain engagement so that they feel stimulated, and timely feedback is provided to patients.

Future research should highlight the advantages of quality of care, improved decision-making, better communication, and patient autonomy.

**Table 6:** What else is needed for their routine implementation?

Study			Contribution
**Systematic reviews**			
	Scheibe et al [26], 2020	Five European programs were identified. In practice, it often needed to be clarified whether the studies identified provided valuable information or crucial aspects, particularly those relating to costs, necessary preconditions, handling of the data collected, and how to enable comparisons. Its application is still limited to a few centers, usually with variations in data collection that do not allow comparison of the centers’ results. The recommendations cover essential aspects, such as the selection of the measure, choice of target patients, timing of assessment, and scoring or reporting techniques. However, it must be made clear whether these recommendations are adhered to in practice and feasible in routine care. This knowledge is imperative to derive an implementation strategy that fits each specific situation, such as in clinical settings, country policies, and stakeholders’ objectives.	
	Liao et al [32], 2021	More experience is needed for its routine application, undoubtedly because of the quality of the articles investigated and the lack of methodology in their reporting, which leads to more studies in their implementation.	
	Bouazza et al [33], 2017	For data comparison to being meaningful and reliable, it must be corrected sufficiently to account for differences in caseness. Further research is needed to support the cost-effectiveness of using PROMs^a^ in clinical practice.	
	Meirte et al [35], 2020	Issues to remember are privacy protection, the substantial initial financial investment, and the exclusion of specific populations. For example, patients may be unwilling or unable to fill out ePROMs^b^ because of older age, disease progression, or computer illiteracy. In addition, some patients need access to the internet, do not have technological devices, or are unfamiliar with them. These disadvantages and barriers should be considered when implementing a digital data collection tool in any population.	
**Qualitative studies**			
	Aiyegbusi et al [25], 2021	Participants felt that completing the questionnaires at home, well before clinic appointments, meant that patients could do so in the comfort of their own homes, without the stress associated with being in the clinic. It also had the advantage of allowing patients more time to think about their health and their responses so the clinic could prepare in advance what to address in the consultation. A decision must be made about who provides proxy data for these patient groups. One transplant patient receiving immunosuppression treatment raised the hygiene issue of iPads being provided for broader use in the clinic.	
	Girgis et al [30], 2022	In the context of research, well-integrated ePROM systems are acceptable and feasible to implement with improved patient and health care system outcomes, including patient-provider communication, patient-provider communication, patient satisfaction, health-related quality of life, chemotherapy compliance, earlier detection of relapse in patients with lung cancer, reduced emergency department visits, and improved cancer survival. However, more research is needed to implement them.	
	Brunelli et al [34], 2022	The systematic collection of PROMs is not widely implemented in routine oncology practice for individual patient care. Difficulty in changing established work practices, the lack of time, and the fear of the negative impact on the patient-physician relationship are the leading causes of its limited use.	
**Factors for implementation**			
	Crockett et al [24], 2021	One of the key learnings from our experience is that maintaining engagement with physicians and patients alike is vital to developing an effective ePROM service.	
	Convill et al [31], 2022	Future research may consider the optimal interval between ePROM collection that best facilitates smoking cessation. In addition, delineating the most likely period of smoking relapse after a lung cancer diagnosis may help clinicians provide specific smoking cessation information during this period.	
**Presentation of experiences**			
	Al Sayah et al [27], 2021	Despite significant advances in research and implementation of PROMs in real-world settings in many countries worldwide, more evidence and guidance are needed to properly implement and use PROMs data. Standards for selecting, collecting, interpreting, and reporting PROMs data with other clinical or administrative data sets are essential to ensure the meaningful use of these data for clinical care and policy decision-making.	
	Carlton et al [28], 2020	The authors propose that careful consideration is given to what specific measures to include, as there appears to be a mismatch between the PROMs measures selected and what physicians may address during the consultation. In addition, they recommend that more attention is paid to introducing patients to follow-up based on PROMs to clarify expectations.	
	Nordan et al [29], 2018	More knowledge of a patient’s initial health status and improvement following the medical or surgical intervention would help to understand necessary care pathways and treatment outcomes better, which is often absent from our current health care processes. Although PROMs are not new, the ability to electronically collect, report, and use data has become more relevant in recent years.	

^a^PROMs: patient-reported outcome measures.

^b^ePROMs: electronic patient-reported outcome measures.

## Discussion

### Principal Findings

ePROMs are digital tools that enable patients to self-report their health status and outcomes. They can be used in various health care settings, including clinical trials, routine care, and population health management. They are designed to register patient-generated data that can be used to supplement clinical paper data and provide a broader picture of a patient’s health status.

These tools are typically web-based or mobile apps that patients can use to complete questionnaires or surveys regarding their symptoms, quality of life, and other health-related topics. Some examples of ePROMs are questionnaires to evaluate the pain, fatigue, or functional status of patients with chronic diseases; quality of life questionnaires for patients with cancer; and evaluations of outcomes after surgical intervention. These surveys measure patients’ perceptions and experiences of their health and treatment, rather than relying on clinical observations or testing. They can also be used to monitor the side effects of treatment or evaluate patient satisfaction.

Institutions and their digitization policies can act as both barriers and facilitators for implementing and using ePROMs. Some of the institutional barriers are a lack of infrastructure and resources to support the use of ePROMs, such as adequate internet connectivity and computer equipment, which can be especially detrimental for patients who live in rural or remote areas; limited technical expertise that may struggle to implement and maintain ePROMs; resistance to change and hesitation to adopt new technologies; costs for the initial implementation and subsequent maintenance, especially if the cost of purchasing or leasing hardware and software is taken into account; and data security concerns, mainly hacking and wrongfully accessing information provided by ePROMs, which can be a barrier to implementation. Implementing ePROMs has also proved more difficult in some medical specialties than in others. In some cases, this was due to the physician’s reluctance to use the International Consortium for Health Outcomes Measurement rule sets [37]. In addition, the history of some employees with previous attempts to implement and collect ePROMs created barriers to the success of this measurement [38].

Nevertheless, there are also several institutional facilitators: better funding through the daily development of telemedicine and broader use of information technologies; strong leadership and support from institutional leaders can help to promote the use of ePROMs and overcome resistance to change; having technical expertise in-house or through partnerships with health technology companies; and transparent policies and guidelines to ensure the safe and secure use of ePROMs, which can help to promote their adoption. ePROMs are preferable to paper-based methods because they improve data quality, result in similar or faster completion times and lower costs, and facilitate clinical decision-making and symptom management [39].

The efficacy of ePROMs depends on their use. When ePROMs are used in a specific patient population, their impact on patient outcomes is higher [40]. It is also necessary to provide patient feedback to make the data collected in this way more meaningful to patients. These ePROMs help patients gain autonomy and control over their current health problems, and reassurance that the clinical team is working around the clock to control any occurrence. This method also proved to be more productive and satisfactory for the professionals involved [40]. By storing data in a central database that is easily accessible through the health unit’s technological infrastructure, clinical information is available to the multidisciplinary team in real time and remotely. Thus, it allows clinicians to monitor the clinical evolution of patients even in an outpatient setting.

Not only do ePROMs help clinicians adequately track an individual patient’s outcome to aid treatment decision-making but they can also be used in quality improvement initiatives, including provider benchmarking. For this purpose, ePROMs can be used as performance indicators for health care institutions and organizations [39]. Furthermore, the inclusion of ePROMs as end points in clinical trials is encouraged by the Food and Drug Administration, the European Medicines Agency, and scientific societies, such as the European Society for Medical Oncology.

ePROMs improve clinical communication by allowing structured presentation of data, thereby facilitating their interpretation. This also permits the use of validated scales to assess patients’ symptomatic and functional control. In addition, patients receiving feedback from the system or health professionals after submitting the questionnaire revealed higher satisfaction levels with the team’s follow-up. ePROMs can facilitate communication between patients and health care providers in several ways:

1. Improved patient engagement: ePROMs enable patients to actively participate in their care by self-assessing their symptoms and functional status, increasing patient engagement and empowerment, and helping to identify problems or issues that may not have been identified through traditional clinical assessments.
2. More complete patient data: ePROMs provide health care providers with additional patient-generated data that can supplement standard clinical data and help providers to make more accurate assessments of their patients.
3. Timely assessments: ePROMs allow patients to receive reminders and complete assessments at their convenience, which can be particularly useful for patients with chronic conditions who may have difficulty visiting clinics for regular appointments.
4. Remote monitoring: ePROMs can be used to monitor patients remotely from the comfort of their homes and can be submitted electronically. This can speed up the data collection and allow for more frequent assessments.
5. Real-time feedback: ePROMs provide real-time feedback on a patient’s symptoms and outcomes, which can help health care providers adjust their treatment plans as needed.

Most patients exposed to ePROMs found it easy to learn and use, would recommend it to other patients, and would like to continue using it [41]. However, a small sample of patients still showed reluctance to accept the remote submission of the clinical questionnaires instead of the classic paper-and-pen version. Finally, there are concerns regarding data privacy and security. Time and energy are needed to ensure strict compliance with the committee framework and organizational policies and procedures [15].

Finally, an essential factor that might contribute to improved outcomes for patients with lung cancer in the care pathway is the fact that not only medical or physical problems are monitored but also much attention is paid to psychosocial, spiritual, and financial burden concerns about relatives and issues around palliative care and end-of-life dilemmas. Patients were more open to reporting on psychological issues and asking for psychological support using a remote digital monitoring system than during a face-to-face conversation in the physician’s office. ePROMs also lowered the bar to express concerns about palliative and end-of-life care. This results in earlier palliative interventions, which increase survival and quality of life of patients with lung cancer [12].

Overall, ePROMs can facilitate communication between patients and health care providers by providing complete and timely patient data, enabling remote monitoring, and allowing for more efficient assessments. However, it is essential to remember that ePROMs are not a substitute for clinical assessment and that health care providers should use ePROMs data in conjunction with other data and information sources when making decisions about patient care.

More evidence is required to support the routine application of ePROMs in health care settings. Although ePROMs have been shown to have many potential benefits, there is still a need for more research to demonstrate their effectiveness in real-world settings and to determine the optimal use of ePROMs in different settings and patient populations. In addition, further research is needed to understand how ePROMs can be integrated into clinical practice and how they can be used to improve patient outcomes, including the best ways to collect, analyze, and use the data generated by these tools; to determine the cost-effectiveness of ePROMs; and how they can be used to improve care while controlling costs.

It is also essential to consider that ePROMs are not a one-size-fits-all solution. Choosing the right ePROMs for specific settings, patient populations, and clinical questions is vital to obtain accurate, reliable, and valid results.

### Limitations

Although more evidence is needed to support the routine application of ePROMs, it is also essential to consider that ePROMs can be valuable tools for health care providers to use in specific scenarios. Therefore, one of the most important limitations of this study was the need for more research that shows results in this area, especially in implementing ePROMs in routine health care.

### Conclusions

The systematic collection of remote ePROMs is an effective and valuable strategy for providing real-time clinical feedback to teams. In addition, it provides satisfaction to patients and professionals. They provide a more accurate view of health outcomes and obtain qualified data in real time. In addition, it allows for easier stratification of patients with multiple pathologies. Routine implementation of ePROMs is an effective and valuable strategy to provide real-time clinical feedback to teams, leading to increased satisfaction from both patients and professionals and optimizing better care for patients. Optimizing ePROMs leads to a more accurate view of health outcomes, ensuring quality and real-time patient health monitoring. It also allows stratifying patients based on their morbidity, creating specific follow-ups for their needs. Data privacy and security must be considered when using ePROMs to ensure compliance with the local entities. At least four barriers were identified:

1. Cost: one of the most important barriers to ePROMs. They are generally more expensive than other types of measure, making them impractical for applications that require large amounts of data storage.
2. Complex programming: ePROMs require computer programming, which can be challenging for novice users, and may prevent their use in some institutions.
3. Security: another important drawback of ePROMs is that it is relatively easy to read data, which can compromise confidential information.
4. Further studies are necessary to prove that ePROMs are helpful instruments to help patients and medical physicians manage their decisions and care. Nonetheless, ePROMs should be considered a standard tool in the future of lung cancer treatment, thus enabling a better understanding of new therapies and new patient outcomes.

## Abbreviations

ePROM: electronic patient-reported outcome measure
HRQoL: health-related quality of life
PRISMA-RR: Preferred Reporting Items for Systematic Reviews and Meta-Analyses for rapid reviews
PROM: patient-reported outcome measure

## Data Availability

The data sets used or analyzed during this study are available from the corresponding author upon reasonable request.
